# Development of a Novel Multiplex PCR Method for the Rapid Detection of SARS-CoV-2, Influenza A Virus, and Influenza B Virus

**DOI:** 10.1155/2024/4950391

**Published:** 2024-02-29

**Authors:** Liang Ma, Haoyan Zhu, Yongwei Jiang, Xiaomu Kong, Peng Gao, Yi Liu, Meimei Zhao, Guoxiong Deng, Yongtong Cao

**Affiliations:** Department of Clinical Laboratory, China-Japan Friendship Hospital, Beijing 100029, China

## Abstract

**Objective:**

A sensitive and specific multiplex fluorescence rapid detection method was established for simultaneous detection of SARS-CoV-2, influenza A virus, and influenza B virus in a self-made device within 30 min, with a minimum detection limit of 200 copies/mL.

**Methods:**

Based on the genome sequences of SARS-CoV-2, influenza A virus (FluA), and influenza B virus (FluB) with reference to the Chinese Center for Disease Control and Prevention and related literature, specific primers were designed, and a multiplex fluorescent PCR system was established. The simultaneous and rapid detection of SARS-CoV-2, FluA, and FluB was achieved by optimizing the concentrations of Taq DNA polymerase as well as primers, probes, and Mg^2+^. The minimum detection limits of the nucleic acid rapid detection system for SARS-CoV-2, FluA, and FluB were evaluated.

**Results:**

By optimizing the amplification system, the N enzyme with the best amplification performance was selected, and the optimal concentration of Mg^2+^ in the multiamplification system was 3 mmol/L; the final concentrations of SARS-CoV-2 NP probe and primer were 0.15 *μ*mol/L and 0.2 *μ*mol/L, respectively; the final concentrations of SARS-CoV-2 ORF probe and primer were both 0.15 *μ*mol/L; the final concentrations of FluA probe and primer were 0.2 *μ*mol/L and 0.3 *μ*mol/L, respectively; the final concentrations of FluB probe and primer were 0.15 *μ*mol/L and 0.25 *μ*mol/L, respectively.

**Conclusion:**

A multiplex real-time quantitative fluorescence RT-PCR system for three respiratory viruses of SARS-CoV-2, FluA, and FluB was established with a high amplification efficiency and sensitivity reaching 200 copies/mL for all samples. Combined with the automated microfluidic nucleic acid detection system, the system can achieve rapid detection in 30 minutes.

## 1. Background

The coronavirus disease 2019 (COVID-19) is an emerging acute respiratory infectious disease that has now evolved into a major global public health event. It has been demonstrated that early detection, reporting, and isolation of the disease can effectively contain the spread and dissemination of coronavirus [1–3]. During winter, however, it becomes more difficult to diagnose COVID-19 with increasing transmission of other respiratory viruses with similar symptoms.

COVID-19 and influenza are both respiratory infectious diseases, which can be transmitted through droplets and contact, and the early symptoms of both are similar and difficult to distinguish, such as fever, dry cough, and sore throat [4–6].

COVID-19 is caused by a type B coronavirus (SARS-CoV-2) with a genome of around 30 kb [7–9], which is a single-stranded RNA (+) virus belonging to beta-coronavirus and is capable of infecting both the avian and human species. Influenza is caused by RNA viruses of the *Orthomyxoviridae* family with genomes of about 14 kb, including influenza A (FluA), influenza B (FluB), and influenza C (FluC) viruses [9, 10]. Influenza A and B viruses may cause regional or even large-scale epidemics. Influenza viruses and SARS-CoV-2 can invade the epithelial cells of the upper respiratory tract, and virions are spread by large droplets produced by infected individuals when they cough and sneeze, which leads to the invasion of the epithelial cells of the upper respiratory tract [11, 12].

The Diagnosis and Treatment Protocol of COVID-19 (Trial Version 8) requires that the suspected COVID-19 cases should be diagnosed by methods including rapid antigen testing and multiplex PCR nucleic acid testing to distinguish from influenza virus infection. In addition, the combined assay for SARS-CoV-2, FluA, and FluB is able to detect coinfections. A study involving 93 cases found that 50% of SARS-CoV-2 infections were coinfected with FluA/B, which may lead to earlier organ damage in patients with critical conditions [2]. Concurrent tests for COVID-19, FluA, and FluB not only reduce the number of tests required for patients but also allow a timely clinical treatment plan for coinfected patients.

Differential diagnosis of SARS-CoV-2 and influenza viruses will be helpful in establishing appropriate strategies for public health and patient management, especially in the diagnosis of suspected cases, critical cases, and in the identification of potential outbreak risks. Adding influenza detection to COVID-19 assays can effectively shorten the test time and improve the efficiency of available equipment, personnel, and reagents, which is cost-effective for the containment of the COVID-19 pandemic. A rapid test for SARS-CoV-2, FluA, and FluB is needed during the prevalent seasons of respiratory viruses to control the pandemic and allow timely diagnosis and treatment for patients.

The most cost-effective preventive and control measure in the face of various emerging infectious diseases is to establish rapid and accurate nucleic acid molecular diagnostic methods, which are based on fully automated and integrated molecular diagnostic systems. The fully automated and integrated molecular diagnostic system can automatically complete the entire process of testing, including sample lysis, nucleic acid extraction, nucleic acid rinsing, nucleic acid elution, gene amplification (PCR), and real-time fluorescence quantitative detection, and can realize the “samples-in, result-out” [13].

The integrated molecular diagnostic system has several advantages over the common PCR method: it does not require a nucleic acid extractor or PCR instrument to be used in conjunction with the test, but only one integrated instrument. Compared with the ordinary PCR method, the integrated molecular diagnostic system has multiple advantages: it is not necessary to match the equipment such as nucleic acid extraction instruments, PCR instruments, and other equipment, and only one integrated instrument can be detected; the operator simply needs to add the sample to the kit and insert it into the instrument for testing, without requiring any additional steps during the testing process, thereby optimizing time and effort efficiency; fully closed automated experimental process can avoid sample cross-contamination and environmental pollution to maximize the protection of the operator's safety. Nowadays, many organizations are actively carrying out the development and research of fully automated and integrated molecular diagnostic systems [14–19].

In order to achieve rapid nucleic acid detection of SARS-CoV-2, FluA, and FluB, we designed a rapid multiplex real-time fluorescence PCR (RT-PCR) assay based on our self-made prototype Fully Automated Nucleic Acid Amplification Testing System (FANAT-1) for simultaneous detection of SARS-CoV-2, FluA, and FluB.

## 2. Materials and Methods

### 2.1. Materials

#### 2.1.1. Samples

*(1) Nucleic Acid Samples*. Nucleic acid samples include SARS-CoV-2, FluA, FluB, and Psrp Synthetic RNA (Sangon Biotech).

*(2) Clinical Samples*. Clinical samples include SARS-CoV-2, H1N1, H3N2, H7N9, H5N1, H1N1 (2009) of FluA, the Victoria lineage, and Yamagata lineage of FluB; all clinical samples or viral cultures of different subtypes were obtained from China-Japan Friendship Hospital.

#### 2.1.2. Instruments and Reagents

This study adopted the prototype Fully Automated Nucleic Acid Amplification Testing System (FANAT-1), as well as primers and probes (Sangon Biotech) and nucleic acid extraction kit (QIAGEN). The basic PCR system contains High-Affinity HotStart Taq and TIANSeq M-MLV (defined as N enzyme, TIANGEN Biotech, Cat nos. ET108 and NG212, separately), MgCl_2_ (25 mM) (Sangon Biotech, Cat no. B601193), One-Step PrimeScript III (defined as A enzyme, Takara Biotech, Cat no. RR601A), Anstart One-Step RT-PCR Mix (heat-labile UDG) (defined as E enzyme, Fapon Biotech, Cat no. MD013P), and 5 × Neoscript RT Premix-UNG (Probe qRT-PCR) (DG) (defined as F enzyme, Biori Biotech, Cat no. FM5254).

### 2.2. Methods

#### 2.2.1. Design of Primers and Probes

The conserved sequences of the SARS-CoV-2 N gene, ORF1ab gene, FluA M gene, and FluB NS gene were selected as amplification targets, and specific primers and fluorescent probes were designed to detect the sample RNA through the change of fluorescent signals (Table 1).

#### 2.2.2. Establishment of PCR System

A conventional PCR procedure and a rapid amplification procedure were set up in the experiment for the amplification of the SARS-CoV-2 N gene and FluA M gene to evaluate the effects of enzymes from different manufacturers on the amplification results and to select the best Taq DNA polymerase for the rapid amplification system.

1. Reagent preparation: four systems of reagent configurations were carried out according to Table 2 with the reaction system of 50 *μ*L.
2. Amplification template: it includes SARS-CoV-2, FluA, and FluB synthetic RNA.
3. The amplification reagents were prepared and tested separately on the prototype Fully Automated Nucleic Acid Amplification Testing System (PFANAT-1), with the number of parallel tests being *N* = 3 for each condition before calculating the average Ct values and selecting the best rapid amplification system. The amplification procedure was configured according to Table 3. Amplification procedure 1 is the best solution after considering the amplification conditions of the four manufacturers, which is defined as the conventional amplification procedure. Amplification procedure 2 is a fast amplification program optimized for amplification time, which is defined as the rapid amplification procedure.

#### 2.2.3. Amplification System Optimization

1. Ten different Mg^2+^ concentrations (final concentrations of 0 mmol/L, 1 mmol/L, 1.5 mmol/L, 2 mmol/L, 2.5 mmol/L, 3 mmol/L, 3.5 mmol/L, 4 mmol/L, 5 mmol/L, and 6 mmol/L) were set up, and synthetic RNA from FluA was taken as the test sample to assess the effect of different Mg^2+^ concentrations on the amplification.
2. The target gene probes of SARS-CoV-2 ORF, SARS-CoV-2 N, influenza A, and influenza B were set with four concentrations each (final concentrations of 0.05 *μ*mol/L, 0.1 *μ*mol/L, 0.15 *μ*mol/L, and 0.2 *μ*mol/L, respectively), and eight concentrations were set for the primers (final concentrations of 0.05 *μ*mol/L, 0.1 *μ*mol/L, 0.15 *μ*mol/L, 0.2 *μ*mol/L, 0.25 *μ*mol/L, 0.3 *μ*mol/L, 0.35 *μ*mol/L, and 0.4 *μ*mol/L); synthetic RNA using SARS-CoV-2, FluA, FluB, and negative control were used as the test samples to examine the effect of different concentrations of probes and primers on the amplification.

#### 2.2.4. Validation of the Minimum Detection Limit

The dilution gradient of *in vitro*-transcribed RNA of SARS-CoV-2, FluA, or FluB at concentrations calibrated by digital PCR was prepared to determine Ct values and create a standard curve; the concentrations of virus cultures were calibrated by the standard curve. Then, serial dilution samples were detected and a 90% positive detection rate was calibrated by the four-parameter fitting algorithm to determine the lowest detection limit, and the clinical samples were diluted to 200 copies/mL using a sample preservation solution to confirm the lowest detection limit. The tests were repeated 20 times under the optimized conditions, and the positive detection rate was obtained based on the tests of three samples of different sources of each virus tested with three different batches of kits. Among them, FluA included five subtypes, H1N1, H3N2, H7N9, H5N1, and H1N1 (2009), and FluB included two subtypes, Victoria and Yamagata.

## 3. Results

### 3.1. Effects of Enzymes on SARS-CoV-2 and FluA Tests

**Figure 1:**
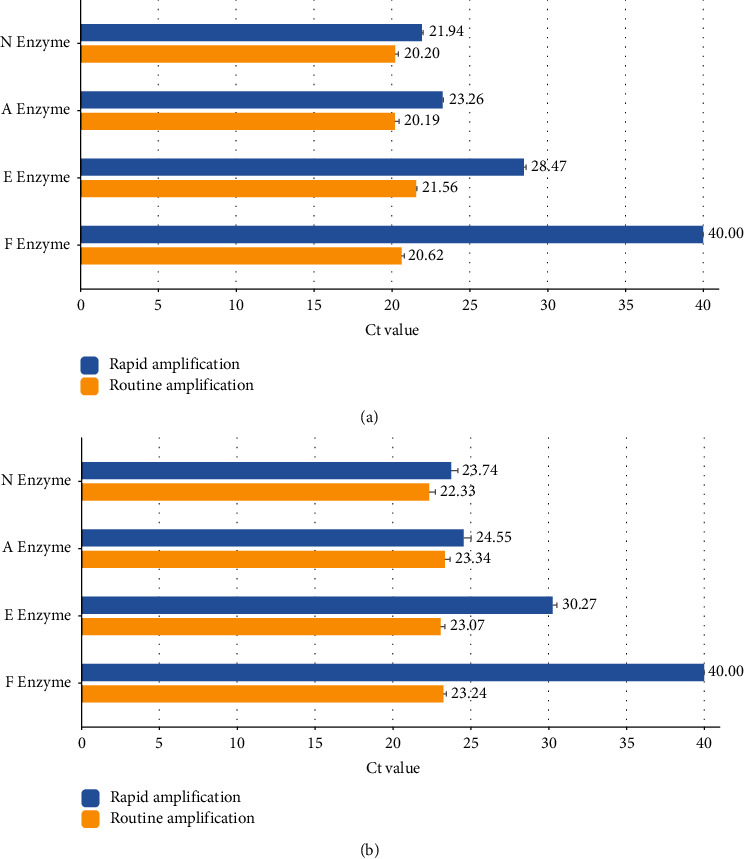
(a) Comparison of the amplification efficiency of the SARS-CoV-2 N gene by different enzymes in the conventional amplification procedure and the rapid amplification procedure. (b) Comparison of the amplification efficiency of FluA by different enzymes in the conventional amplification procedure and the rapid amplification procedure.

The results are shown in Figure 1, which indicates that F enzyme is unable to achieve rapid amplification, E enzyme Ct is delayed, and A enzyme and N enzyme can lead to rapid amplification, with N enzyme working the best.

### 3.2. Results of FluA Tests at Different Mg^2+^ Concentrations

**Figure 2:**
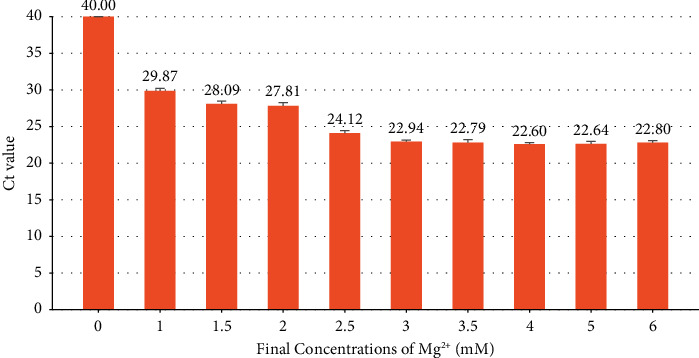
FluA test results at different Mg^2+^ concentrations.

The Ct value of FluA decreased with the rise of Mg^2+^ concentration, and there was no significant difference when the concentration of Mg^2+^ was at 3 mM or above. Therefore, 3 mM was determined as the optimal concentration of Mg^2+^ considering that excessive concentration would lead to nonspecific amplification (Figure 2).

### 3.3. Test Results of Different Concentrations of Primer Probes

The Ct value of SARS-CoV-2 N decreases when both primer and probe concentrations are increased, so the final concentration of N probe is set at 0.15 *μ*M and the final concentration of primer can be 0.2 *μ*M since the effect of primer is close at the concentrations ranging from 0.05 *μ*M to 0.4 *μ*M (Table 4); the Ct value of SARS-CoV-2 ORF increases when both primer and probe concentrations are increased, so the final concentration of ORF probe is set at 0.15 *μ*M and the final concentration of primer can be 0.15 *μ*M since the effect of primer is desirable at the concentrations ranging from 0.15 *μ*M to 0.25 *μ*M (Table 4). The Ct value of FluA decreases when both primer and probe concentrations are increased, so the final concentration of FluA probe is set at 0.2 *μ*M and the final concentration of primer can be 0.2 *μ*M since the effect of primer is desirable at the concentrations ranging from 0.15 *μ*M to 0.4 *μ*M (Table 4); the Ct value of FluB decreases when both primer and probe concentrations are increased, and therefore the final concentration of FluB probe is set at 0.15 *μ*M and the final concentration of primer can be 0.25 *μ*M since the effect of primer is desirable at the concentrations ranging from 0.15 *μ*M to 0.4 *μ*M (Table 4).

### 3.4. Comparison of Results between Multiplex Systems and Single Systems

In order to optimize the amplification system, amplification tests were performed using the synthetic RNA of SARS-CoV-2, FluA, and FluB, with Psrp RNA (fragment sequences derived from Arabidopsis genomes) as the internal control. Every assay had parallel tests of 3 samples, and the results showed no significant difference in amplifications between the multiplex system and the single-weight system (Table 5).

### 3.5. Results of Minimum Detection Limit

The standard curve of the transcribed RNA dilution gradient for SARS-CoV-2, FluA, or FluB is shown in Supplementary Table 1 and Supplementary Figure 1. Using the formula obtained from the standard curve, the concentrations of the clinical samples or viral cultures can be calculated, which are shown in Table 6.

**Figure 3:**
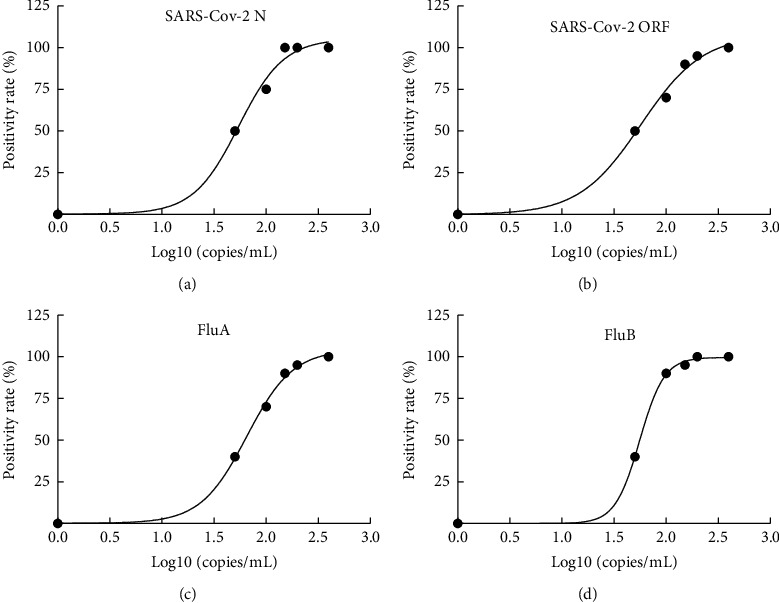
(a) The four-parameter fitting curve of serial dilutions SARS-CoV-2 N gene. (b) The four-parameter fitting curve of serial dilutions SARS-CoV-2 ORF gene. (c) The four-parameter fitting curve of serial dilutions FluA. (d) The four-parameter fitting curve of serial dilutions FluB.

The serial dilutions detection data of each target (SARS-CoV-2, FluA, and FluB) are shown in Supplementary Table 2. The lowest detection limit of the SARS-CoV-2 N gene was 131.14 copies/mL calculated by the following formula: *y* = 0.1969 + 105.2031/(1 + 10 ^ ((1.736-*x*) *∗* 2.006)) from the four-parameter fitting curve (Figure 3(a)). The lowest detection limit of the SARS-CoV-2 ORF gene was 173.47 copies/mL calculated by the following formula: *y* = −0.1358 + 107.8358/(1 + 10 ^ ((1.758-*x*) *∗* 1.469)) from the four-parameter fitting curve (Figure 3(b)). The lowest detection limit of FluA was 166.11 copies/mL calculated by the following formula: *y* = 0.1509 + 104.14911/(1 + 10 ^ ((1.814-*x*) *∗* 1.964)) from the four-parameter fitting curve (Figure 3(c)). The lowest detection limit of FluB was 102.16 copies/mL calculated by the following formula: *y* = −0.0246 + 99.6946/(1 + 10 ^ ((1.747-*x*) *∗* 3.694)) from the four-parameter fitting curve (Figure 3(d)).

As revealed by the results of the three batches of different detection reagents, 200 copies/mL for the clinical samples or viral cultures of SARS-CoV-2, FluA, and FluB all met the requirements of 95%–100% positive detection rate, as shown in Table 7 and Supplementary Table 3. So, the minimum detection limits of SARS-CoV-2, FluA, and FluB can be set to 200 copies/mL.

## 4. Discussion

In response to respiratory infectious diseases, early and rapid diagnosis can control the development of the disease as early as possible and reduce the number of critical patients. However, the premise of rapid diagnosis is to ensure the sensitivity and accuracy of the detection. Therefore, designing a reaction system to ensure rapid and effective amplification of nucleic acid is the core of this study. Finally, a multiplex fluorescence RT-PCR assay was designed in this study to establish and optimize a multiplex amplification system for COVID-19, influenza A, and influenza B to achieve the differential diagnosis of COVID-19 and influenza in 30 minutes.

This study proved that the enzymes from different manufacturers could affect the amplification results, and N enzymes could achieve rapid amplification through experiments. The detection principle is that the DNA polymerase with 5′∼3′ DNA exonuclease activity will degrade the probe when it meets the fluorescence-labeled probe bound to the template strand during the PCR extension, resulting in the release of fluorescence to be detected by the real-time quantitative PCR instrument [20]. On Taq DNA polymerase, the polymerase active region and the 5′∼3′ DNA exonuclease active region are found in different structural domains [21], and these two active regions work together to initiate the synthesis of new DNA strands while cleaving the fluorescent probe and releasing the signal. Among the reactions of polymerization and exocytosis, the less efficient reaction directly determines the efficiency of DNA amplification and the release of fluorescent signals. As reported, the Taq polymerase does not degrade the entire probe, and the degraded part is about 5–12 bp from the 5′ ends of the probe; the undegraded probe may participate in the subsequent PCR cycles, so as to inhibit the release of the fluorescent signal; the degradation of the probe is closely associated with the intensity of the Taq enzyme's exonuclease activity [22, 23]. In the case of the limited amount of enzyme (0.38, 0.19 U/reaction), once the Taq polymerase with the same polymerase activity is added to the reaction, the enzyme with higher exonuclease activity shows higher amplification efficiency; when Taq polymerase with the same exonuclease activity is added, the amplification efficiency is basically similar even though the polymerase activity is different. The abovementioned results demonstrate that the exonuclease reaction is a key step for rate control, and the rate is crucial to the efficiency of DNA amplification.

Due to the dependence of enzyme activity on Mg^2+^ concentration in the PCR reaction system, the absence of Mg^2+^ will lead to enzyme inactivation, and the enzyme activity will be inhibited when the concentration of Mg2+ is high. In the study of optimizing the isothermal amplification reaction system, Mg^2+^ concentration in the system has a significant effect on the amplification efficiency [24]. In addition, the divalent cations also affect the dissociation temperature and annealing temperature of the primer and template hybrid. Therefore, the concentration of Mg^2+^ in the system will affect the amplification efficiency. In this study, it was found that the amplification efficiency was the highest and the expansion speed was the fastest when the Mg^2+^ concentration was 3 mmol/L in the multiple amplification system.

The final concentrations of primers and probes for the three respiratory virus assays were determined through the optimization of concentrations of primer and probe in the amplification system. The final concentrations of SARS-CoV-2 NP probe and primer were 0.15 *μ*mol/L and 0.2 *μ*mol/L, respectively; the final concentrations of SARS-CoV-2 ORF probe and primer were both 0.15 *μ*mol/L; the final concentrations of FluA probe and primer were 0.2 *μ*mol/L and 0.3 *μ*mol/L, respectively; the final concentrations of FluB probe and primer were 0.15 *μ*mol/L and 0.25 *μ*mol/L, respectively. The detection of the three viruses using the multiplex assay system constructed in this study did not show a difference from those of the single system amplification, and the minimum detection limits of the present study for SARS-CoV-2, FluA, and FluB could all reach 200 copies/mL. Relevant research results show that the minimum detection limit of single nucleic acid detection of respiratory pathogens (such as SARS-CoV-2) is mostly in the range of 250–1000 copies/mL, and the amplification time is mostly more than 1 h. Although some studies claim that the minimum detection limit of the method can reach 200 copies/mL or lower, the amplification time is close to 2 h [25–29].

In summary, by optimizing the amplification conditions and using a self-made device that integrates sample lysis, nucleic acid extraction, nucleic acid purification, multiplex fluorescent PCR, and result analysis, this study achieves the purpose of 30 min rapid detection without additional operation and the minimum detection limit of 200 copies/mL. The assay can be used for clinical rapid differential diagnosis, contributing to the combat against the SARS-CoV-2 pandemic as well as the seasonal influenza.

## Figures and Tables

**Table 1:** Primers, probe-target genes, and sequence information.

Virus	Primer and probe	Sequence	Modification
SARS-CoV-2^a^	CoV-ORF-F	CCCTGTGGGTTTTACACTTAA	
	CoV-ORF-P	CCGTCTGCGGTATGTGGAAAGGTTATGG	5′Texas RED, 3′BHQ2
	CoV-ORF-R	ACGATTGTGCATCAGCTGA	
	CoV-NP-F	GGGGAACTTCTCCTGCTAGAAT	
	CoV-NP-P	TTGCTGCTGCTTGACAGATT	5′FAM, 3′BHQ1
	CoV-NP-R	CAGACATTTTGCTCTCAAGCTG	

FluA^b^	FluA-F	TAAAGACAAGACCAATCCTGTCACC	
	FluA-P	ACGCTCACCGTGCCCAGTGAGCGA	5′CY5, 3′BHQ2
	FluA-R	TCCCATTTAGGGCATTTTGGACAAAGC	

FluB^c^	FluB-F	AAAGATGGCCATCGGATCCTC	
	FluB-P	AAAGCCAATTCGAGCAGCTGAAACTG	5′CY5.5, 3′BHQ2
	FluB-R	GCTCTTGACCAAATTGGGAT	

Psrp^d^	Psrp-F	GTCCCTTCATCGTCGCTG	
	Psrp-P	CACCGTTGCTGTTTTCCTTATCGGTTACGC	5′HEX, 3′BHQ1
	Psrp-R	GGCGGTTTGTCAAGCTGAT	

^a^The primer sequences are from “technical guidelines for laboratory testing of novel coronavirus pneumonia” (https://www.chinacdc.cn/jkzt/crb/zl/szkb_11803/jszl_11815/202003/W020200309540843062947.pdf). ^b^The primer sequences have been modified from “National Technical Guidelines for Influenza Surveillance (2017 edition),” (https://ivdc.chinacdc.cn/cnic/zyzx/jcfa/201709/P020170930331067634607.pdf). ^c^The primer sequences have been modified from “Mokkapati, Anupama., Brown, Bradley., Jones, Robert. 2019, methods of detecting influenza, United States, CEPHEID (Sunnyvale, CA, US), 10480036,” (https://www.freepatentsonline.com/10480036.html). ^d^The primer sequences have been designed based on Arabidopsis genome sequences.

**Table 2:** Basic PCR system.

Basic system	N	A	E	F
Composition	Volume (*μ*L)	Volume (*μ*L)	Volume (*μ*L)	Volume (*μ*L)
DNA polymerase	0.5	25	0.75	2
RT enzyme mix	2.5	25	0.4	2
RNase inhibitor	2.5	25	0.5	10
Mg^2+^(25 mM)	3	25	16	10
Reaction buffer	9	25	16	10
Upstream primer (10 *μ*M)	1	1	1	1
Downstream primer (10 *μ*M)	1	1	1	1
Probe (10 *μ*M)	0.5	0.5	0.5	0.5
Sample	5	5	5	5
Enzyme-free sterile water	Fill in up to 50 *μ*L	Fill in up to 50 *μ*L	Fill in up to 50 *μ*L	Fill in up to 50 *μ*L

**Table 3:** Configuration of different amplification procedures.

Reaction phase	Temperature	Duration			
		Amplification procedure 1 (conventional amplification procedure)		Amplification procedure 2 (rapid amplification procedure)	
Degradation U-templates	25°C	10 min		2 min	

Reverse transcription	50°C	15 min		3 min	

Pre-denaturation	95°C	5 min		30 s	

Denaturation	95°C	15 s	40 cycles	3 s	40 cycles
Annealing and extension	60°C	30 s		5 s	

	Total duration	∼1 h 30 min		∼30 min	

**Table 4:** Ct values under different concentrations of primers and probes.

		Primer concentration (*μ*M)							
		0.05	0.1	0.15	0.2	0.25	0.3	0.35	0.4
SARS-CoV-2 NP probe concentration (*μ*M)	0.05	33.24	32.97	33.34	33.22	33.08	33.20	33.04	33.18
	0.10	32.29	32.26	32.33	32.28	32.39	32.20	32.20	32.22
	0.15	32.01	31.86	32.15	31.98	32.17	31.94	32.06	31.97
	0.20	32.50	32.09	32.00	32.02	31.98	32.20	31.75	32.11

SARS-CoV-2 ORF probe concentration (*μ*M)	0.05	34.14	34.04	34.92	36.63	35.54	39.21	N/A	N/A
	0.10	34.20	33.21	33.84	34.98	35.39	37.09	39.82	N/A
	0.15	33.19	32.24	33.86	33.73	34.09	36.70	39.31	N/A
	0.20	33.72	32.62	35.90	35.14	34.31	35.76	39.69	N/A

FluA M probe concentration (*μ*M)	0.05	31.01	30.69	30.62	30.71	30.61	30.76	30.81	31.07
	0.10	30.55	30.27	30.02	30.18	29.98	29.73	29.93	29.98
	0.15	30.25	30.02	29.67	29.71	29.45	29.57	29.60	29.75
	0.20	30.54	29.91	29.62	29.69	29.61	29.11	29.40	29.63

FluB NS probe concentration (*μ*M)	0.05	33.68	33.29	32.66	33.20	32.33	32.88	32.47	32.60
	0.10	33.07	32.77	32.54	32.72	32.67	32.21	32.25	32.76
	0.15	32.97	33.38	32.46	32.53	32.01	32.53	32.33	32.60
	0.20	34.03	32.55	32.63	32.68	32.62	32.98	33.02	32.87

**Table 5:** Ct values of the multiplex PCR system and single PCR system.

High concentration	Multiplex		Single		ΔCt
	Mean	SD	Mean	SD	
ORF	30.34	0.1	30.79	0.14	−0.45
N	29.77	0.12	29.46	0.05	0.31
FluA	34.14	0.26	33.7	0.36	0.44
FluB	33.97	0.34	33.57	0.30	0.40
Psrp	29.66	0.30	29.96	0.31	−0.30

**Table 6:** The concentrations of the clinical samples or viral cultures of SARS-CoV-2, FluA, and FluB.

Virus	Subtype	Sample	Concentration (copies/mL)
SARS-CoV-2	—	CoV-2-1	1.69*E* + 07
		CoV-2-2	5.86*E* + 09
		CoV-2-3	1.70*E* + 06

FluA	H1N1	A11-1	1.01*E* + 06
		A11-2	4.68*E* + 06
		A11-3	1.20*E* + 06
	H3N2	A32-1	1.91*E* + 09
		A32-2	1.28*E* + 05
		A32-3	2.54*E* + 06
	H7N9	A79-1	5.82*E* + 08
		A79-2	1.71*E* + 09
		A79-3	6.35*E* + 08
	H1N1 (2009)	A2009-1	4.25*E* + 04
		A2009-2	3.49*E* + 05
		A2009-3	1.98*E* + 04
	H5N1	A51-1	6.17*E* + 09
		A51-2	2.83*E* + 09
		A51-3	2.97*E* + 09

FluB	Victoria	BV-1	1.23*E* + 06
		BV-2	4.85*E* + 05
		BV-3	1.47*E* + 05
	Yamagata	BY-1	4.91*E* + 05
		BY-2	5.68*E* + 07
		BY-3	3.64*E* + 05

**Table 7:** Positive detection rate for viral culture tests of 200 copies/mL for SARS-CoV-2, FluA, and FluB.

Virus	Subtype	Sample	Target	Batch 1 (%)	Batch 2 (%)	Batch 3 (%)
SARS-CoV-2	—	Sample 1	ORF	100	95	100
		N	100	100	100
		Sample 2	ORF	100	100	95
		N	100	95	100
		Sample 3	ORF	100	100	100
		N	95	100	100

FluA	H1N1	Sample 1	M	100	100	95
		Sample 2	M	95	100	100
		Sample 3	M	100	95	100
	H3N2	Sample 1	M	95	100	100
		Sample 2	M	100	100	100
		Sample 3	M	100	95	100
	H7N9	Sample 1	M	100	100	100
		Sample 2	M	100	95	100
		Sample 3	M	95	100	100
	H1N1 (2009)	Sample 1	M	100	100	100
		Sample 2	M	100	100	95
		Sample 3	M	100	100	100
	H5N1	Sample 1	M	100	95	100
		Sample 2	M	100	100	100
		Sample 3	M	100	100	100

FluB	Victoria	Sample 1	NS	100	100	95
		Sample 2	NS	100	100	100
		Sample 3	NS	95	100	100
	Yamagata	Sample 1	NS	100	95	100
		Sample 2	NS	100	100	100
		Sample 3	NS	95	100	100

## Data Availability

The data that support the findings of this study are available from the corresponding author upon reasonable request.

## Supplementary Materials

Supplementary Materials Supplementary Table 1: raw data for the standard curve of each target (SARS-CoV-2, FluA, and FluB). Supplementary Figure 1: the standard curve of SARS-CoV-2, FluA, and FluB. Supplementary Table 2: raw data for the serial dilutions detection of each target (SARS-CoV-2, FluA, and FluB). Supplementary Table 3: raw data for viral culture tests of 200 copies/mL for SARS-CoV-2, FluA, and FluB.
