# Supplementary material for: Development of a Novel Multiplex PCR Method for the Rapid Detection of SARS-CoV-2, Influenza A Virus, and Influenza B Virus
Source: Int J Anal Chem. 2024 Feb 29;2024:4950391. doi: 10.1155/2024/4950391 (PMC10919977; doi:10.1155/2024/4950391)
Supplement: Supplementary Materials — Supplementary Table 1: raw data for the standard curve of each target (SARS-CoV-2, FluA, and FluB). Supplementary Figure 1: the standard curve of SARS-CoV-2, FluA, and FluB. Supplementary Table 2: raw data for the serial dilutions detection of each target (SARS-CoV-2, FluA, and FluB). Supplementary Table 3: raw data for viral culture tests of 200 copies/mL for SARS-CoV-2, FluA, and FluB. [file 4950391.f1.docx]

**Supplementary table 1**

Raw data for the standard curve of each target (SARS-Cov-2, FluA and FluB).

| target | concentration (copies) | Log10 (copies） | Repeat 1 | Repeat 2 | Repeat 3 | Ct-Mean | SD |
| --- | --- | --- | --- | --- | --- | --- | --- |
| SARS-COV-2 synthetic RNA-N gene | 2.4E+05 | 5.38 | 23.52 | 23.59 | 23.53 | 23.55 | 0.04 |
|  | 4.8E+04 | 4.68 | 25.81 | 25.78 | 25.59 | 25.73 | 0.12 |
|  | 9.6E+03 | 3.98 | 28.01 | 28.06 | 28.01 | 28.03 | 0.03 |
|  | 1.9E+03 | 3.28 | 29.99 | 30.24 | 30.33 | 30.18 | 0.18 |
|  | 3.8E+02 | 2.58 | 32.86 | 32.79 | 32.30 | 32.65 | 0.31 |
| FluA synthetic RNA | 5.6E+06 | 6.75 | 16.15 | 16.25 | 16.28 | 16.22 | 0.07 |
|  | 5.6E+05 | 5.75 | 20.16 | 20.07 | 20.07 | 20.10 | 0.05 |
|  | 5.6E+04 | 4.75 | 23.47 | 23.48 | 23.36 | 23.44 | 0.07 |
|  | 5.6E+03 | 3.75 | 27.21 | 27.39 | 27.26 | 27.29 | 0.09 |
|  | 5.6E+02 | 2.75 | 31.01 | 30.70 | 30.83 | 30.85 | 0.15 |
|  | 5.6E+01 | 1.75 | 34.21 | 34.16 | 34.07 | 34.15 | 0.07 |
| FluB synthetic RNA | 3.4E+06 | 6.54 | 15.00 | 15.03 | 15.08 | 15.04 | 0.04 |
|  | 3.4E+05 | 5.54 | 18.82 | 18.52 | 18.60 | 18.65 | 0.16 |
|  | 3.4E+04 | 4.54 | 22.40 | 22.38 | 22.44 | 22.40 | 0.03 |
|  | 3.4E+03 | 3.54 | 25.78 | 25.69 | 25.77 | 25.75 | 0.05 |
|  | 3.4E+02 | 2.54 | 29.81 | 29.54 | 29.60 | 29.65 | 0.14 |
|  | 3.4E+01 | 1.54 | 33.02 | 33.01 | 32.55 | 32.86 | 0.27 |

**Supplementary Fig.1**

The standard curve of SARS-Cov-2, FluA and FluB.

**Supplementary table 2**

Raw data for The serial dilutions detection data of each target (SARS-Cov-2, FluA and FluB).

| Virus | Target | Sample concentration (copies/mL) | 1 | 2 | 3 | 4 | 5 | 6 | 7 | 8 | 9 | 10 | 11 | 12 | 13 | 14 | 15 | 16 | 17 | 18 | 19 | 20 | Positive detection rate |
| --- | --- | --- | --- | --- | --- | --- | --- | --- | --- | --- | --- | --- | --- | --- | --- | --- | --- | --- | --- | --- | --- | --- | --- |
| SARS-Cov-2 | N | 0 | N/A | N/A | N/A | N/A | N/A | N/A | N/A | N/A | N/A | N/A | N/A | N/A | N/A | N/A | N/A | N/A | N/A | N/A | N/A | N/A | 0% |
| SARS-Cov-2 | N | 50 | 38.56 | 38.04 | N/A | 38.47 | 38.34 | N/A | 38.70 | 37.97 | N/A | N/A | 39.54 | N/A | 38.01 | 38.01 | N/A | N/A | N/A | 39.19 | N/A | N/A | 50% |
| SARS-Cov-2 | N | 100 | N/A | 39.03 | 38.43 | 38.76 | N/A | 38.52 | N/A | 38.41 | 38.61 | 38.01 | 37.75 | 38.11 | N/A | 38.27 | 38.07 | 38.24 | 37.91 | N/A | 38.28 | 37.88 | 75% |
| SARS-Cov-2 | N | 150 | 37.34 | 37.74 | 37.74 | 37.33 | 38.19 | 37.56 | 37.22 | 37.52 | 38.33 | 38.07 | 38.61 | 37.12 | 38.07 | 36.40 | 37.41 | 36.80 | 38.31 | 38.90 | 37.57 | 37.91 | 100% |
| SARS-Cov-2 | N | 200 | 36.95 | 36.58 | 39.19 | 35.93 | 37.22 | 38.63 | 37.24 | 36.75 | 37.22 | 36.62 | 36.54 | 37.32 | 35.91 | 37.27 | 36.81 | 36.57 | 37.69 | 37.18 | 36.87 | 37.30 | 100% |
| SARS-Cov-2 | N | 400 | 35.94 | 36.12 | 36.11 | 35.99 | 36.00 | 36.23 | 37.64 | 35.82 | 35.91 | 36.25 | 37.53 | 35.33 | 36.05 | 37.14 | 36.90 | 37.24 | 36.04 | 37.11 | 36.36 | 39.19 | 100% |
| SARS-Cov-2 | ORF | 0 | N/A | N/A | N/A | N/A | N/A | N/A | N/A | N/A | N/A | N/A | N/A | N/A | N/A | N/A | N/A | N/A | N/A | N/A | N/A | N/A | 0% |
| SARS-Cov-2 | ORF | 50 | 37.25 | 35.50 | N/A | 36.97 | 37.21 | N/A | 38.09 | 37.61 | N/A | N/A | 37.14 | N/A | 35.17 | 37.69 | N/A | N/A | N/A | 38.14 | N/A | N/A | 50% |
| SARS-Cov-2 | ORF | 100 | N/A | 38.10 | 36.26 | 36.35 | N/A | N/A | N/A | 36.88 | 37.86 | 36.97 | 37.96 | 36.30 | N/A | 37.00 | 36.43 | 37.97 | 35.98 | N/A | 37.20 | 37.92 | 70% |
| SARS-Cov-2 | ORF | 150 | 36.89 | 37.51 | 36.19 | 36.80 | 36.41 | 37.25 | 34.86 | 36.32 | 38.04 | 37.21 | 37.34 | 37.22 | 36.94 | N/A | 37.20 | 35.97 | 36.89 | 37.47 | 35.80 | 36.77 | 95% |
| SARS-Cov-2 | ORF | 200 | 36.27 | 35.98 | 36.65 | 34.98 | 34.99 | 37.41 | 36.51 | 35.91 | 35.92 | 34.26 | 36.35 | 36.65 | 34.83 | 36.34 | 35.52 | 37.45 | 36.56 | 36.24 | 35.62 | 37.23 | 100% |
| SARS-Cov-2 | ORF | 400 | 35.89 | 34.65 | 36.50 | 35.56 | 34.23 | 34.39 | 36.48 | 35.81 | 36.54 | 36.72 | 36.51 | 35.63 | 35.37 | 37.55 | 36.65 | 36.63 | 35.44 | 34.56 | 35.69 | 36.56 | 100% |
| FluA | M | 0 | N/A | N/A | N/A | N/A | N/A | N/A | N/A | N/A | N/A | N/A | N/A | N/A | N/A | N/A | N/A | N/A | N/A | N/A | N/A | N/A | 0% |
| FluA | M | 50 | N/A | N/A | N/A | 38.93 | N/A | 39.58 | 38.13 | N/A | N/A | 38.09 | 37.84 | N/A | 38.15 | N/A | 37.83 | 39.55 | N/A | N/A | N/A | N/A | 40% |
| FluA | M | 100 | N/A | N/A | 36.99 | 36.46 | N/A | 37.37 | 38.10 | N/A | 37.48 | 39.71 | N/A | 37.15 | 37.04 | 37.11 | 38.03 | 36.78 | 36.92 | 36.55 | N/A | 37.85 | 70% |
| FluA | M | 150 | 36.32 | 36.61 | 35.41 | 36.45 | 37.20 | 37.32 | 36.59 | 35.66 | 37.11 | N/A | 37.06 | N/A | 36.86 | 36.39 | 36.24 | 37.66 | 37.44 | 36.97 | 37.10 | 36.27 | 90% |
| FluA | M | 200 | 36.38 | 35.60 | 36.20 | 36.23 | 36.20 | N/A | 34.78 | 36.10 | 35.95 | 35.97 | 37.30 | 37.45 | 37.31 | 36.26 | 35.35 | 35.33 | 36.25 | 36.40 | 36.37 | 35.88 | 95% |
| FluA | M | 400 | 34.94 | 34.84 | 35.56 | 36.15 | 34.98 | 35.34 | 34.92 | 34.99 | 36.90 | 36.94 | 35.83 | 37.49 | 35.85 | 36.60 | 35.01 | 34.91 | 34.79 | 34.37 | 36.33 | 35.02 | 100% |
| FluB | NS | 0 | N/A | N/A | N/A | N/A | N/A | N/A | N/A | N/A | N/A | N/A | N/A | N/A | N/A | N/A | N/A | N/A | N/A | N/A | N/A | N/A | 0% |
| FluB | NS | 50 | N/A | 38.08 | N/A | N/A | N/A | 39.37 | 37.70 | N/A | N/A | 38.18 | N/A | 38.02 | N/A | 38.79 | N/A | 37.43 | 39.84 | N/A | N/A | N/A | 40% |
| FluB | NS | 100 | 36.65 | N/A | 37.02 | 36.86 | 37.54 | 38.57 | 37.45 | 37.51 | 37.21 | 37.17 | 37.26 | N/A | 36.33 | 36.75 | 36.82 | 37.19 | 36.76 | 36.79 | 37.46 | 35.73 | 90% |
| FluB | NS | 150 | 37.13 | 35.43 | 37.59 | 36.12 | 35.97 | 36.09 | 35.99 | 37.14 | 36.66 | 36.87 | 36.66 | 36.29 | 37.50 | 36.47 | 38.05 | 36.53 | 36.90 | 36.58 | N/A | 37.16 | 95% |
| FluB | NS | 200 | 35.60 | 36.92 | 37.01 | 36.40 | 36.41 | 36.39 | 34.98 | 36.16 | 36.39 | 36.54 | 37.07 | 36.41 | 38.06 | 36.34 | 36.00 | 35.67 | 35.79 | 35.61 | 37.08 | 36.58 | 100% |
| FluB | NS | 400 | 36.98 | 35.18 | 37.09 | 35.50 | 35.11 | 34.95 | 35.09 | 36.52 | 35.42 | 34.38 | 35.23 | 35.05 | 35.97 | 36.71 | 34.59 | 35.21 | 34.64 | 35.18 | 35.17 | 36.53 | 100% |

**Supplementary table 3**

Raw data for viral culture tests of 200 copies/mL for SARS-Cov-2, FluA and FluB.

| Virus | Subtype | Sample | Target | Batch | 1 | 2 | 3 | 4 | 5 | 6 | 7 | 8 | 9 | 10 | 11 | 12 | 13 | 14 | 15 | 16 | 17 | 18 | 19 | 20 | Positive detection rate |
| --- | --- | --- | --- | --- | --- | --- | --- | --- | --- | --- | --- | --- | --- | --- | --- | --- | --- | --- | --- | --- | --- | --- | --- | --- | --- |
| SARS-Cov-2 | / | Sample 1 | ORF | 1 | 38.86 | 36.41 | 37.66 | 35.87 | 35.93 | 39.18 | 36.37 | 39.27 | 36.19 | 35.93 | 35.99 | 35.76 | 39.08 | 36.36 | 37.27 | 36.14 | 39.32 | 36.2 | 36.19 | 38.98 | 100% |
| SARS-Cov-2 | / | Sample 1 | ORF | 2 | 37.99 | 36.72 | 37.02 | N/A | 36.79 | 36.32 | 35.32 | 35.5 | 35.32 | 35.97 | 36.06 | 35.5 | 36.54 | 35.97 | 35.6 | 35.98 | 37.17 | 37.65 | 35.83 | 36.06 | 95% |
| SARS-Cov-2 | / | Sample 1 | ORF | 3 | 33.3 | 36.54 | 34.89 | 36.44 | 37.17 | 36.32 | 37.65 | 35.83 | 37.92 | 35.35 | 33.3 | 35.45 | 37.53 | 35.68 | 36.74 | 35.57 | 36.34 | 37 | 34.89 | 35.72 | 100% |
| SARS-Cov-2 | / | Sample 1 | N | 1 | 38.13 | 38.91 | 36.3 | 38.64 | 36.71 | 39.22 | 36.13 | 38.85 | 35.9 | 38.08 | 36.49 | 37.22 | 38.72 | 38.4 | 35.97 | 36.92 | 39.08 | 36.96 | 37.86 | 39.42 | 100% |
| SARS-Cov-2 | / | Sample 1 | N | 2 | 36.38 | 37.03 | 35.93 | 37.55 | 38.34 | 38.14 | 37.46 | 36.21 | 37.46 | 36.59 | 36.17 | 36.21 | 36.03 | 36.59 | 36.54 | 35.88 | 38.35 | 37.64 | 36.1 | 36.17 | 100% |
| SARS-Cov-2 | / | Sample 1 | N | 3 | 33.12 | 36.03 | 37.41 | 37.4 | 38.35 | 37.55 | 37.64 | 36.1 | 36.8 | 36.06 | 33.12 | 36.2 | 37.28 | 36.64 | 37.3 | 35.93 | 37.47 | 35.78 | 37.41 | 37.4 | 100% |
| SARS-Cov-2 | / | Sample 2 | ORF | 1 | 36.74 | 36.52 | 35.74 | 35.78 | 33.63 | 35.61 | 34.88 | 34.92 | 37.58 | 36.56 | 37.52 | 34.24 | 34.43 | 36.16 | 33.52 | 37.05 | 35.34 | 36.33 | 36.45 | 36.83 | 100% |
| SARS-Cov-2 | / | Sample 2 | ORF | 2 | 37.97 | 37.91 | 37.99 | 35.49 | 38.18 | 37.12 | 36.69 | 36.96 | 37.79 | 38.18 | 37.12 | 36.69 | 35.1 | 36.29 | 36.96 | 37.79 | 35.1 | 36.29 | 36.79 | 36.32 | 100% |
| SARS-Cov-2 | / | Sample 2 | ORF | 3 | 37.4 | 35.52 | 37.12 | 37.28 | 35.66 | 36.39 | 35.48 | 37.12 | 36.4 | 35.54 | 37.27 | 37.19 | 37.62 | 37.13 | 35.82 | N/A | 37.22 | 37.24 | 37.11 | 37.56 | 95% |
| SARS-Cov-2 | / | Sample 2 | N | 1 | 37.03 | 36.29 | 37.12 | 38.53 | 35.65 | 35.83 | 35.74 | 36.2 | 36.93 | 37.03 | 37.93 | 35.37 | 35.29 | 35.85 | 34.1 | 38.54 | 36.56 | 35.72 | 36.02 | 37.43 | 100% |
| SARS-Cov-2 | / | Sample 2 | N | 2 | 38.31 | N/A | 37.91 | 35.32 | 39.43 | 37.42 | 37.27 | 38.3 | 37.35 | 39.43 | 37.42 | 37.27 | 36.58 | 36.82 | 38.3 | 37.35 | 36.58 | 36.82 | 38.91 | 38.14 | 95% |
| SARS-Cov-2 | / | Sample 2 | N | 3 | 36.41 | 36.12 | 35.25 | 36.04 | 36.36 | 37.1 | 36.06 | 35.82 | 37.4 | 37.66 | 35.98 | 35.09 | 35.74 | 36.54 | 36.09 | 36.75 | 37.05 | 36.9 | 36.72 | 36.75 | 100% |
| SARS-Cov-2 | / | Sample 3 | ORF | 1 | 35.89 | 34.62 | 34.51 | 35.79 | 35.43 | 35.78 | 34.29 | 34.18 | 36.18 | 35.93 | 35.9 | 34.96 | 34.8 | 34.64 | 33.95 | 34.82 | 35.2 | 34.71 | 34.15 | 33.95 | 100% |
| SARS-Cov-2 | / | Sample 3 | ORF | 2 | 37.3 | 37.38 | 36.05 | 37.4 | 36.1 | 37.04 | 37.35 | 36.54 | 35.93 | 37.49 | 37.38 | 35.7 | 37.4 | 36.23 | 35.9 | 37.28 | 36.48 | 37.46 | 35.6 | 37.27 | 100% |
| SARS-Cov-2 | / | Sample 3 | ORF | 3 | 37.56 | 35.84 | 36.11 | 35.27 | 37.25 | 37.52 | 36.05 | 37.16 | 37.21 | 36.65 | 36.23 | 36.02 | 34.89 | 37.31 | 37.27 | 36.55 | 37.76 | 36.03 | 34.83 | 37.47 | 100% |
| SARS-Cov-2 | / | Sample 3 | N | 1 | 37 | 34.85 | 35.55 | 36.06 | N/A | 37.11 | 35.44 | 35.42 | 36.76 | 36.25 | 36.66 | 36.32 | 37.6 | 35.81 | 35.7 | 35.69 | 36.07 | 35.33 | 36.49 | 35.49 | 95% |
| SARS-Cov-2 | / | Sample 3 | N | 2 | 35.87 | 36.74 | 36.14 | 36.35 | 36.73 | 35.98 | 36.25 | 36.1 | 36.81 | 35.29 | 37.22 | 36.48 | 36.74 | 36.04 | 36.38 | 36.17 | 37.36 | 37.08 | 36.62 | 35.85 | 100% |
| SARS-Cov-2 | / | Sample 3 | N | 3 | 37 | 35.98 | 37.01 | 36.46 | 35.94 | 36.48 | 35.96 | 35.92 | 37.65 | 37.3 | 36.14 | 36.31 | 36.43 | 36.02 | 36.26 | 36.35 | 37.41 | 36.56 | 36.21 | 36.21 | 100% |
| FluA | H1N1 | Sample 1 | M | 1 | 35 | 39.89 | 34.38 | 33.6 | 33.53 | 35.08 | 34.67 | 35.37 | 35.11 | 35.41 | 37.04 | 35.51 | 35.5 | 36.51 | 36.38 | 35.84 | 36.86 | 35.59 | 39.36 | 37.07 | 100% |
| FluA | H1N1 | Sample 1 | M | 2 | 35.2 | 35.38 | 35.4 | 35.58 | 37.18 | 36.06 | 35.07 | 36.43 | 35.91 | 37.28 | 37.48 | 36.78 | 36.61 | 39.01 | 36.51 | 36.22 | 36.2 | 36.8 | 38.75 | 38.07 | 100% |
| FluA | H1N1 | Sample 1 | M | 3 | 34.73 | 38.46 | 34.96 | N/A | 34.55 | 34.97 | 34.61 | 37.82 | 38.2 | 35.59 | 35.26 | 35.98 | 35.07 | 36.14 | 35.28 | 35.37 | 35.17 | 33.76 | 36.75 | 34.83 | 95% |
| FluA | H1N1 | Sample 2 | M | 1 | N/A | 37.09 | 34.6 | 35.51 | 35.11 | 35.46 | 34.5 | 35.13 | 34.85 | 36.29 | 37.11 | 36.79 | 35.51 | 33.88 | 36.52 | 35.74 | 37.73 | 36.58 | 34.88 | 35.26 | 95% |
| FluA | H1N1 | Sample 2 | M | 2 | 35.15 | 35.14 | 35.11 | 35.7 | 35.29 | 36.01 | 35.64 | 36.93 | 36.29 | 36.97 | 35.73 | 35.58 | 35.11 | 34.31 | 34.34 | 34.1 | 33.09 | 34.02 | 34.47 | 35.25 | 100% |
| FluA | H1N1 | Sample 2 | M | 3 | 35.78 | 34.81 | 35.05 | 36.71 | 35.17 | 33.92 | 36.46 | 34.54 | 35.65 | 39.48 | 34.36 | 35.23 | 34.01 | 34.27 | 35.15 | 33.56 | 35.84 | 35.63 | 35.85 | 37.86 | 100% |
| FluA | H1N1 | Sample 3 | M | 1 | 35.46 | 37.66 | 37.79 | 35.85 | 37.38 | 36.65 | 35.96 | 35.43 | 35.59 | 35.5 | 36.03 | 36.9 | 35.87 | 35.68 | 37.36 | 37.11 | 35.32 | 36.51 | 36.68 | 37.77 | 100% |
| FluA | H1N1 | Sample 3 | M | 2 | 35.89 | 36.92 | 36.66 | 37.38 | 35.18 | 35.58 | 35.5 | 35.76 | 36.63 | 35.27 | 37.64 | 35.73 | 37.4 | 35.29 | 36.88 | 37.27 | N/A | 37.08 | 36.04 | 36.09 | 95% |
| FluA | H1N1 | Sample 3 | M | 3 | 37.11 | 37.35 | 37.6 | 37.09 | 37.04 | 36.37 | 36.36 | 35.71 | 37.09 | 36.52 | 37.02 | 37.54 | 35.56 | 35.75 | 36.03 | 35.21 | 37.23 | 36.89 | 35.92 | 36.52 | 100% |
| FluA | H3N2 | Sample 1 | M | 1 | 35.88 | 35.31 | 33.3 | 35.42 | 35.6 | 34.59 | 35.85 | 37.11 | 37.75 | 36.49 | N/A | 37.97 | 37.08 | 35.19 | 35.19 | 34.54 | 34.3 | 38.83 | 34 | 32.86 | 95% |
| FluA | H3N2 | Sample 1 | M | 2 | 35.81 | 37.18 | 37.9 | 37.3 | 37.87 | 38.01 | 37.17 | 36.79 | 34.2 | 38.93 | 33.21 | 36.96 | 34.38 | 33.07 | 35.92 | 34.02 | 33.86 | 34.67 | 34.56 | 33.98 | 100% |
| FluA | H3N2 | Sample 1 | M | 3 | 35.67 | 36.04 | 34.27 | 36 | 34.6 | 34.59 | 35.71 | 35.81 | 34.76 | 34.41 | 35.35 | 37.68 | 34.84 | 36.77 | 36.77 | 34.92 | 35.75 | 33.26 | 34.01 | 33.56 | 100% |
| FluA | H3N2 | Sample 2 | M | 1 | 36.87 | 36.81 | 36.12 | 35.84 | 36.25 | 37 | 34.88 | 35.26 | 33.59 | 36.81 | 35.51 | 34.95 | 35.85 | 36.92 | 36.36 | 36.75 | 36.24 | 35.34 | 34.61 | 34.21 | 100% |
| FluA | H3N2 | Sample 2 | M | 2 | 34.98 | 34.83 | 36.46 | 35.18 | 36.34 | 35.39 | 35.58 | 35.88 | 35.4 | 35.61 | 36.46 | 37.79 | 37.09 | 35.2 | 34.89 | 35.05 | 34.62 | 35.51 | 34.8 | 35.06 | 100% |
| FluA | H3N2 | Sample 2 | M | 3 | 35.51 | 37.03 | 37.18 | 34.4 | 34.66 | 38.49 | 36.51 | 37.06 | 35.99 | 37.22 | 35.7 | 34.23 | 34.81 | 35.3 | 35.22 | 36.41 | 39.59 | 35.93 | 36.5 | 35.78 | 100% |
| FluA | H3N2 | Sample 3 | M | 1 | 37.74 | 37.28 | 37.3 | 37.55 | 36.83 | 37.32 | 36.52 | 36.01 | 36.75 | 36.8 | 36.62 | 36.04 | 37.54 | 35.55 | 35.35 | 35.9 | 35.14 | 37.15 | 36.77 | 35.73 | 100% |
| FluA | H3N2 | Sample 3 | M | 2 | 35.98 | 35.82 | 37.47 | 37.6 | 35.85 | 37.35 | 36.65 | 35.72 | 35.56 | N/A | 35.69 | 36.42 | 36.74 | 35.92 | 35.57 | 37.22 | 37.08 | 35.82 | 36.55 | 37 | 95% |
| FluA | H3N2 | Sample 3 | M | 3 | 35.52 | 36.34 | 37.54 | 37.29 | 37.45 | 35.69 | 35.52 | 35.57 | 37.13 | 37.81 | 37.62 | 37.02 | 37.26 | 36.32 | 37.88 | 36.94 | 37.51 | 37.47 | 37.37 | 37.74 | 100% |
| FluA | H7N9 | Sample 1 | M | 1 | 37.22 | 36.63 | 37.59 | 37.87 | 37.54 | 37.65 | 37.86 | 37.79 | 37.88 | 35.67 | 37.18 | 35.84 | 36.46 | 35.82 | 37.13 | 37.07 | 37.78 | 35.81 | 36.51 | 36.79 | 100% |
| FluA | H7N9 | Sample 1 | M | 2 | 36.75 | 36.24 | 35.7 | 36.17 | 37.61 | 37.61 | 37.47 | 35.68 | 35.58 | 35.66 | 37.33 | 37.9 | 37.73 | 37.15 | 37.38 | 36.28 | 37.66 | 37.02 | 35.87 | 37.44 | 100% |
| FluA | H7N9 | Sample 1 | M | 3 | 35.34 | 36.12 | 37.62 | 37.26 | 37.35 | 36.62 | 36.15 | 37 | 37.75 | 37.21 | 36.29 | 36.18 | 35.41 | 35.26 | 36.04 | 35.82 | 37.49 | 37.36 | 36.25 | 36.47 | 100% |
| FluA | H7N9 | Sample 2 | M | 1 | 37.88 | 36.16 | 35.9 | 36.03 | 37.49 | 37.81 | 35.97 | 37.35 | 36.81 | 35.69 | 35.5 | 35.66 | 36.1 | 36.44 | 36.78 | 35.68 | 35.47 | 37.26 | 37.18 | 35.24 | 100% |
| FluA | H7N9 | Sample 2 | M | 2 | 35.96 | 37.27 | 35.83 | 35.32 | 36.7 | 36.55 | 37.26 | 35.12 | 35.8 | 35.48 | N/A | 36.55 | 35.43 | 37.49 | 35.81 | 37.66 | 35.45 | 36.9 | 37.18 | 37.65 | 95% |
| FluA | H7N9 | Sample 2 | M | 3 | 36.56 | 36.69 | 37.51 | 37.2 | 37.31 | 37.55 | 36.68 | 37.37 | 36.43 | 35.89 | 36.47 | 36.51 | 36.55 | 37.16 | 37.61 | 35.22 | 35.58 | 36.21 | 35.2 | 37.31 | 100% |
| FluA | H7N9 | Sample 3 | M | 1 | 36.03 | 35.87 | 36.05 | 35.34 | 35.34 | 35.84 | 36.67 | 36.14 | 37.22 | 36.27 | 35.38 | 36.78 | 37.72 | 35.72 | 36.53 | N/A | 36.23 | 37.56 | 37.74 | 37.44 | 95% |
| FluA | H7N9 | Sample 3 | M | 2 | 37.69 | 37.87 | 35.45 | 37.12 | 35.36 | 36.05 | 35.85 | 36.02 | 36.47 | 37.5 | 37.44 | 37.19 | 37.48 | 37.3 | 37.01 | 35.64 | 37.76 | 36.79 | 37.25 | 37.76 | 100% |
| FluA | H7N9 | Sample 3 | M | 3 | 37.92 | 37.23 | 36.3 | 37.18 | 37.86 | 37.48 | 37.6 | 37.68 | 37.5 | 37.56 | 35.93 | 36.93 | 36.16 | 36.53 | 35.8 | 36.74 | 37.1 | 37.17 | 36.17 | 36.64 | 100% |
| FluA | H1N1(2009) | Sample 1 | M | 1 | 36.03 | 38.19 | 35.41 | 35.08 | 35.71 | 37.71 | 35.82 | 35.62 | 37.99 | 38.02 | 38.95 | 35.58 | 35.54 | 38.73 | 37.82 | 38.18 | 35.85 | 35.54 | 35.54 | 36.32 | 100% |
| FluA | H1N1(2009) | Sample 1 | M | 2 | 36.28 | 37.37 | 35.53 | 36.8 | 36.92 | 37.22 | 35.29 | 35.97 | 35.06 | 34.82 | 36.22 | 34.85 | 36.47 | 35.2 | 35.08 | 36.81 | 36.97 | 35.02 | 37.84 | 36.54 | 100% |
| FluA | H1N1(2009) | Sample 1 | M | 3 | 35.96 | 36.13 | 35.85 | 39.97 | 34.8 | 37.45 | 34.6 | 38.31 | 36.18 | 35.61 | 34.83 | 35.52 | 34.4 | 35.36 | 34.37 | 34.57 | 37.16 | 34.11 | 36.32 | 34.82 | 100% |
| FluA | H1N1(2009) | Sample 2 | M | 1 | 36.26 | 37.59 | 37.38 | 37.65 | 35.69 | 35.29 | 35.73 | 37.41 | 37.64 | 37.63 | 36.36 | 37.2 | 36.38 | 37.64 | 36.88 | 35.75 | 37.47 | 37.4 | 37.43 | 35.49 | 100% |
| FluA | H1N1(2009) | Sample 2 | M | 2 | 37.43 | 35.2 | 36.59 | 35.72 | 35.66 | 36.54 | 37.68 | 37.26 | 37.91 | 37.51 | 37.46 | 37.22 | 35.41 | 37.96 | 36.94 | 37.3 | 37.71 | 37.91 | 36.9 | 36.44 | 100% |
| FluA | H1N1(2009) | Sample 2 | M | 3 | 37.46 | 37.92 | 37.66 | 37.79 | 37.83 | 37.73 | 37.68 | 35.67 | 37.22 | 35.95 | 36.4 | 35.83 | 37.19 | 37.2 | 37.94 | 35.95 | 36.46 | 36.8 | 36.24 | N/A | 95% |
| FluA | H1N1(2009) | Sample 3 | M | 1 | 36.02 | 35.38 | 35.58 | 35.74 | 36.38 | 36.16 | 37.19 | 36.23 | 36.09 | 36.7 | 37.4 | 35.58 | 36.64 | 35.27 | 36.65 | 37.54 | 37.91 | 37.31 | 35.14 | 35.83 | 100% |
| FluA | H1N1(2009) | Sample 3 | M | 2 | 37.45 | 37.17 | 37.41 | 36.64 | 36.06 | 36.86 | 37.4 | 37.23 | 36.37 | 36.28 | 35.46 | 35.39 | 35.55 | 35.92 | 37.69 | 37.76 | 36.34 | 36.48 | 36.16 | 37.19 | 100% |
| FluA | H1N1(2009) | Sample 3 | M | 3 | 35.94 | 35.61 | 36.8 | 36.73 | 37.24 | 35.08 | 35.57 | 35.04 | 35.76 | 36.95 | 35.39 | 37.65 | 35.7 | 37.62 | 35.41 | 36.92 | 37.34 | 37.75 | 37.68 | 36.12 | 100% |
| FluA | H5N1 | Sample 1 | M | 1 | 35.21 | 37.27 | 37.36 | 36.06 | 35.34 | 35.52 | 36.57 | 37.31 | 37.28 | 37.04 | 37.36 | 37.27 | 37.06 | 35.81 | 37.72 | 36.93 | 37.33 | 37.63 | 37.88 | 37.6 | 100% |
| FluA | H5N1 | Sample 1 | M | 2 | 36.46 | 37.39 | 37.89 | 37.92 | 37.65 | 37.93 | 37.54 | 37.69 | 35.21 | 36.91 | 35.94 | N/A | 35.71 | 37.01 | 37.13 | 36.49 | 36.11 | 36.43 | 36.95 | 36.62 | 95% |
| FluA | H5N1 | Sample 1 | M | 3 | 36.07 | 36.26 | 35.23 | 35.43 | 35.71 | 36.63 | 36.13 | 37.23 | 36.17 | 35.47 | 36.58 | 37.35 | 35.67 | 36.03 | 35.16 | 36.24 | 37.62 | 37.78 | 37.34 | 36.01 | 100% |
| FluA | H5N1 | Sample 2 | M | 1 | 35.81 | 37.48 | 37.4 | 37.45 | 36.36 | 35.76 | 36.89 | 37.25 | 37.14 | 36.34 | 36.34 | 35.44 | 35.63 | 35.26 | 35.91 | 37.65 | 37.97 | 36.28 | 36.48 | 35.96 | 100% |
| FluA | H5N1 | Sample 2 | M | 2 | 37.12 | 36.26 | 35.65 | 36.91 | 36.48 | 37.13 | 35.97 | 35.54 | 35.45 | 35.76 | 36.46 | 35.27 | 37.79 | 36.02 | 37.72 | 35.3 | 37.04 | 36.99 | 37.77 | 37.72 | 100% |
| FluA | H5N1 | Sample 2 | M | 3 | 35.94 | 36.12 | 35.93 | 37.5 | 37.87 | 35.9 | 37.34 | 36.69 | 35.71 | 35.5 | 35.65 | 35.61 | 36.35 | 36.64 | 35.7 | 35.54 | 37.3 | 37.07 | 37.75 | 36.49 | 100% |
| FluA | H5N1 | Sample 3 | M | 1 | 36.88 | 37.75 | 37.21 | 37.61 | 37.45 | 36.76 | 37.39 | 36.6 | 36.09 | 36.71 | 37.36 | 36.56 | 37.01 | 37.58 | 35.51 | 37.14 | 36 | 35.32 | 37.32 | 37.04 | 100% |
| FluA | H5N1 | Sample 3 | M | 2 | 36.08 | 35.53 | 36.23 | 37.49 | 37.54 | 37.52 | 35.71 | 35.55 | 35.55 | 37.24 | 37.76 | 37.74 | 37.38 | 36.79 | 36.4 | 37.64 | 37.05 | 35.89 | 37.47 | 37.29 | 100% |
| FluA | H5N1 | Sample 3 | M | 3 | 37.77 | 35.44 | 37.47 | 35.37 | 36.49 | 35.82 | 35.53 | 36.41 | 37.36 | 37.15 | 37.94 | 37.57 | 37.33 | 37.14 | 35.71 | 37.67 | 36.98 | 36.98 | 37.81 | 37.89 | 100% |
| FluB | Victoria | Sample 1 | NS | 1 | 36.56 | 37.99 | 37.69 | 35.82 | 37.6 | 36.46 | 35.32 | 36.61 | 36.47 | 37.28 | 34.45 | 37.08 | 35.87 | 38 | 36.16 | 35.8 | 36.51 | 36.85 | 39.29 | 34.56 | 100% |
| FluB | Victoria | Sample 1 | NS | 2 | 33.46 | 37.03 | 35.09 | 34.43 | 37.34 | 35.55 | 36.86 | 38.43 | 37.01 | 36.6 | 38.27 | 37.48 | 38.43 | 35.97 | 39.32 | 35.73 | 38.49 | 35.63 | 38.21 | 38.39 | 100% |
| FluB | Victoria | Sample 1 | NS | 3 | 35.39 | 35.73 | 35.79 | 36.04 | 34.09 | 35.29 | 36.62 | 34.49 | 35.14 | 35.23 | 35.86 | N/A | 34.65 | 35.16 | 36.22 | 34.04 | 34.88 | 34.67 | 36.04 | 36.18 | 95% |
| FluB | Victoria | Sample 2 | NS | 1 | 39.05 | 38.07 | 36.98 | 38.43 | 36.12 | 37.3 | 39.7 | 36.98 | 35.74 | 36.37 | 36.71 | 34.72 | 39.71 | 37.84 | 38.99 | 36.87 | 36.87 | 35.86 | 37.36 | 35.55 | 100% |
| FluB | Victoria | Sample 2 | NS | 2 | 38.89 | 35.27 | 36.77 | 36.05 | 36.19 | 36.61 | 38.96 | 36.59 | 37.78 | 37.76 | 36.82 | 36.16 | 36.34 | 39.19 | 36.63 | 36.43 | 33.81 | 36.38 | 38.84 | 35.21 | 100% |
| FluB | Victoria | Sample 2 | NS | 3 | 35.97 | 37.26 | 35.94 | 36.72 | 37.72 | 37.46 | 35.13 | 35.72 | 34.82 | 36.61 | 35.93 | 36.34 | 36.81 | 37.07 | 36.28 | 36.63 | 36.18 | 38.01 | 37.79 | 35.74 | 100% |
| FluB | Victoria | Sample 3 | NS | 1 | 36.48 | 36.27 | 35.94 | 35.7 | 35.8 | N/A | 36.95 | 35.21 | 35.97 | 36.5 | 36.52 | 35.37 | 36.25 | 37.83 | 36.99 | 35.51 | 37.49 | 37.09 | 36.66 | 35.69 | 95% |
| FluB | Victoria | Sample 3 | NS | 2 | 36.99 | 35.52 | 37.48 | 37.1 | 36.66 | 35.67 | 35.76 | 36.07 | 35.52 | 35.75 | 36.06 | 36.12 | 36.88 | 37.16 | 35.01 | 35.3 | 35.01 | 36.76 | 35.82 | 36.87 | 100% |
| FluB | Victoria | Sample 3 | NS | 3 | 35.01 | 35.33 | 35.01 | 36.76 | 35.83 | 36.87 | 36.48 | 36.24 | 35.93 | 35.72 | 35.81 | 35.57 | 36.94 | 35.19 | 35.97 | 36.49 | 36.51 | 35.39 | 36.65 | 37.82 | 100% |
| FluB | Yamagata | Sample 1 | NS | 1 | 36.93 | 36.5 | 36.35 | 38.92 | 37.63 | 36.72 | 37.67 | 39.74 | 35.93 | 35.95 | 36.39 | 34.95 | 34.72 | 35.58 | 35.26 | 35.51 | 35.8 | 34.78 | 35.16 | 35.09 | 100% |
| FluB | Yamagata | Sample 1 | NS | 2 | 35.78 | 36.09 | 35.51 | 35.75 | 36.03 | 36.14 | 36.89 | 37.14 | 35.8 | 35.32 | 35.03 | 36.75 | 35.82 | 36.84 | 36.48 | 36.26 | 35.94 | N/A | 35.79 | 35.57 | 95% |
| FluB | Yamagata | Sample 1 | NS | 3 | 36.09 | 37.94 | 36.47 | 35.95 | 36.09 | 37.21 | 37.48 | 37.82 | 36.54 | 36.35 | 35.61 | 38.52 | 33.89 | 35.75 | 37.62 | 37.31 | 36.02 | 37.51 | 38.9 | 36.85 | 100% |
| FluB | Yamagata | Sample 2 | NS | 1 | 35.62 | 35.88 | 35.47 | 37.38 | 36.68 | 34.72 | 38.19 | 34.73 | 36.07 | 37.63 | 35.98 | 35.87 | 35.68 | 37.42 | 35.11 | 35.59 | 34.38 | 35.41 | 35.31 | 36.04 | 100% |
| FluB | Yamagata | Sample 2 | NS | 2 | 36.33 | 35.65 | 37.12 | 37.19 | 35.72 | 35.12 | 35.83 | 34.48 | 35.6 | 36.9 | 36.6 | 36.06 | 36.66 | 36.39 | 36.92 | 37.19 | 36.08 | 34.99 | 38.66 | 36.97 | 100% |
| FluB | Yamagata | Sample 2 | NS | 3 | 37.22 | 38.83 | 35.29 | 38.51 | 36.46 | 36.16 | 37.51 | 34.64 | 36.9 | 36.57 | 34.56 | 35.7 | 36.74 | 34.59 | 36.08 | 35.99 | 34.49 | 36.7 | 38.41 | 36.41 | 100% |
| FluB | Yamagata | Sample 3 | NS | 1 | 36.9 | 37.17 | 35 | 35.83 | 35.02 | 36.78 | 35.83 | 36.85 | 36.5 | 36.24 | 35.95 | 35.7 | 35.8 | 35.58 | 36.94 | N/A | 35.96 | 36.51 | 36.51 | 35.38 | 95% |
| FluB | Yamagata | Sample 3 | NS | 2 | 36.95 | 35.21 | 35.97 | 36.5 | 36.53 | 35.37 | 36.67 | 37.83 | 37.08 | 35.8 | 37.49 | 37.08 | 36.63 | 35.67 | 35.76 | 36.09 | 35.02 | 35.77 | 36.03 | 36.13 | 100% |
| FluB | Yamagata | Sample 3 | NS | 3 | 33.18 | 34.36 | 34.85 | 34.73 | 33.93 | 35.75 | 34.04 | 36.63 | 34.2 | 37.15 | 36.41 | 36.49 | 34.82 | 35.92 | 37.62 | 34.99 | 36.07 | 35.9 | 37.71 | 36.69 | 100% |
